# Comparison between Local Excision and Radical Resection for the Treatment of Rectal Cancer in ypT0-1 Patients: An Analysis of the Clinicopathological Factors and Survival Rates

**DOI:** 10.3390/cancers13194823

**Published:** 2021-09-27

**Authors:** Soo Young Oh, In Ja Park, Young IL Kim, Jong-Lyul Lee, Chan Wook Kim, Yong Sik Yoon, Seok-Byung Lim, Chang Sik Yu, Jin Cheon Kim

**Affiliations:** Asan Medical Center, Department of Colon and Rectal Surgery, University of Ulsan College of Medicine, Seoul 05505, Korea; soyooh@naver.com (S.Y.O.); illie8246@gmail.com (Y.I.K.); iamleejong@amc.seoul.kr (J.-L.L.); crscwkim@amc.seoul.kr (C.W.K.); yoonys@amc.seoul.kr (Y.S.Y.); sblim@amc.seoul.kr (S.-B.L.); csyu@amc.seoul.kr (C.S.Y.); jckim@amc.seoul.kr (J.C.K.)

**Keywords:** rectal cancer, chemoradiotherapy, local excision, radical resection, recurrence-free survival, overall survival

## Abstract

**Simple Summary:**

Rectal cancer with good clinical response after preoperative chemoradiotherapy (PCRT) have shown favorable outcomes. As favorable oncologic outcomes in patients with good response to PCRT emerged, it dawned on many colorectal surgeons that the rectum can be saved without compromising the prognosis of rectal cancer. However, evidence was not sufficient yet, and it was necessary to determine the factors associated with oncologic outcomes after local excision. The purpose of our study was to compare the oncologic outcomes between local excision and radical resection in ypT0-1 patients and verify the oncologic safety of local excision. Our study provides valid surgery principles by analyzing the prognostic factors of each strategy, further reinforcing the evidence of rectum sparing treatment for rectal cancer patients.

**Abstract:**

Tumors with good response to preoperative chemoradiotherapy have a favorable prognosis, and these findings raise interest in rectum-sparing strategies. This study aimed to compare the oncologic outcome between local excision and radical resection in ypT0-1 patients and to analyze prognostic factors. Patients with primary rectal cancer diagnosed with ypT0-1 after PCRT followed by either radical resection (RR) or local excision (LE) between 2005 and 2014 were included in this study (LE = 78, RR = 442). Clinicopathologic features, recurrence-free survival (RFS), and OS were analyzed. There was no statistically significant difference in the RFS and OS between the LE and RR groups. Clinical T stage (cT3-4) before PCRT was related to RFS and in the LE group (*p* = 0.022). Lymph node metastasis (HR: 4.884, 95% confidence interval: 2.451–9.732, *p* < 0.001) in the final pathology was the only factor associated with RFS, showing a statistically significant difference in the RR group. Lymph node metastasis and age were associated with OS in the RR group. This study confirms the oncologic feasibility of LE in ypT0-1 rectal cancer after PCRT. Additionally, careful patient selection with higher accuracy modalities should be updated to improve treatment outcomes of LE.

## 1. Introduction

Preoperative chemoradiotherapy (PCRT) has led to a new era of treatment for rectal cancer. This may be attributed to the significant improvements in local control, better compliance with the regimen, and a greater chance in preserving the anal sphincter of patients with low rectal cancers by downsizing as well as down-staging the tumor instead of subjecting them to abdominoperineal resection.

The response to PCRT varies from complete response to no response, and it is well known that patients with a good response to PCRT or rectal cancer had better long-term outcomes than those with a poor response [1,2]. Many studies showed a better long-term outcome in the group with good pathologic response and confirm a strong positive prognostic value of downgraded tumor after PCRT [1,2,3,4,5].

Currently, radical resection (RR) of the rectum with total mesorectal excision is a standard treatment for rectal cancer treated with PCRT, which has been attributed to curative surgical treatment and is also a way to fully evaluate the final pathologic stage of rectal cancer, including the profiling of sufficient lymph nodes (LNs) during staging. However, it is associated with significant morbidity, both immediate and late. A considerable proportion of patients require permanent stoma, report sexual dysfunction, and experience bowel and bladder dysfunction or low anterior syndrome, which affects long-term quality of life [6,7].

Therefore, favorable oncologic outcomes in patients with clinical good response raised interest in rectum-sparing strategies for patients with good responses to PCRT by suggesting local excision (LE) or a watch-and-wait approach [8,9,10,11]. In addition to reports of favorable oncologic outcomes in patients with good response to PCRT, further studies [9,11] comparing oncologic outcomes between LE and RR in patients with good response to PCRT also reported no significant difference between the two excisional strategies.

Despite LE being an appealing alternative organ-sparing strategy to surgeons, there is still concern for suboptimal staging. In an oncologically negative perspective regarding LE, limited evaluation of mesorectal LNs still precludes definitive tumor staging. Moreover, in cases of recurrence after LE, local recurrence in the previously resected site is difficult to manage. Moreover, extra-luminal lymph node recurrence is even more difficult to diagnose than luminal recurrence, and salvage surgery after recurrence often shows R1 resection [12,13].

In addition, although continuously developing, the use of imaging modalities, such as computed tomography (CT), magnetic resonance imaging (MRI), endorectal ultrasonography (EUS), and positron emission tomography (PET), in clinical staging to confirm the indication of LE has limited use of evaluation, especially in tissues with radiation-induced fibrosis, and is particularly worse in accuracy of nodal staging in the setting of T1 and T2 cancers [14,15,16,17]. The risk of residual positive LNs in the irradiated mesorectum is reported to vary between 0% and 17% even with PCR of the primary tumor, thus, the need for evaluation of mesorectal LNs [1,16,17]. The oncologic soundness of LE has not been sufficiently established for patients with good response after PCRT.

Therefore, this study aimed to compare the oncologic outcome between LE and RR in ypT0-1 patients and provide a valid surgery principle by analyzing the prognostic factors of the strategies.

## 2. Materials and Methods

### 2.1. Patients

**Figure 1:**
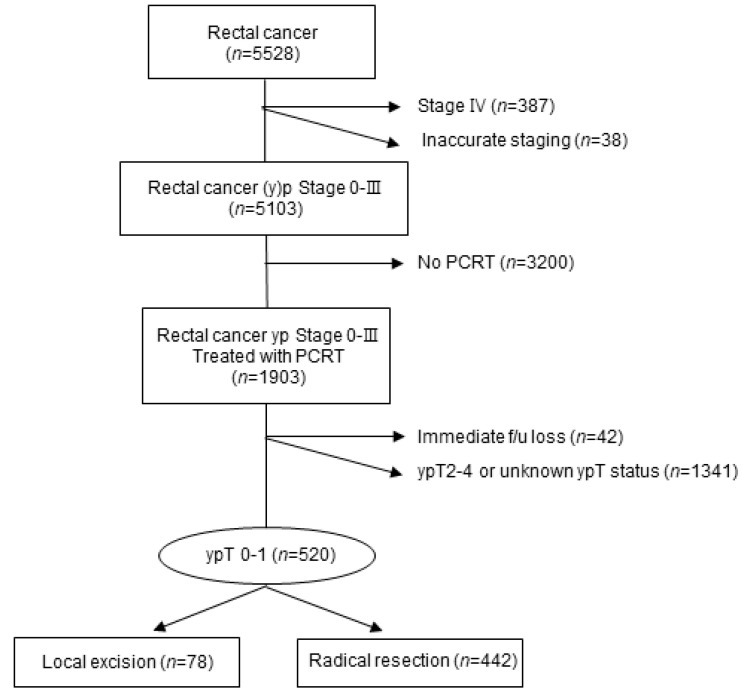
CONSORT diagram for patient inclusion. PCRT, preoperative chemoradiotherapy.

Patients who were diagnosed with primary rectal cancer (ypT0-1) after being treated with PCRT followed by either RR or LE with an Eastern Cooperative Oncology Group score of 0–2 at Asan Medical Center, Seoul, Korea, between 2005 and 2014 were included in this study and retrospectively analyzed. Among the 5528 patients diagnosed with rectal cancer at Asan Medical Center (Seoul, Korea), those diagnosed with clinical stage 4 disease or with synchronous metastatic disease (*n* = 387) and those with inaccurate staging (*n* = 38) were excluded, regardless of tumor size. Patients who did not undergo PCRT (*n* = 3200) were also excluded. Although 1903 patients completed all cycles of PCRT, those who were immediately lost to follow-up (*n* = 42) and those diagnosed with ypT2-4 or with unknown ypT status (*n* = 1341) were excluded. A total of 520 patients were diagnosed with ypT0-1; among them, 442 patients received RR and 78 patients underwent LE (Figure 1).

### 2.2. Treatment

Patients with rectal cancers diagnosed with clinical T3 and T4 disease, and those with node-positive tumors that showed a threatened circumferential resection margin of <1 mm on MRI [18] were recommended to receive PCRT. However, patients with less advanced disease underwent PCRT for sphincter preservation in cases of low rectal cancer, presence of severe medical comorbidities, or reluctance to undergo upfront surgery.

The patients received a total dose of 50.0–50.4 Gy of radiotherapy performed five times a week for 5 weeks, with 23 to 25 fractions of local irradiation to the pelvis (1.8–2.0 Gy each) and a boost dose 4.0–5.4 Gy radiation to the primary tumor over 3 days. Concurrent chemotherapy consisted of either two cycles of an intravenous bolus injection of 5-fluorouracil (5-FU, 375 mg/m^2^/day) and leucovorin (20 mg/m^2^/day) (FL) for 3 days during the first and fifth week of radiotherapy or oral administration of capecitabine (825 mg/m^2^) twice daily. Oxaliplatin was used as a combined regimen in some patients.

After completing 4 to 6 weeks of PCRT, all patients were reevaluated through physical examination, abdominopelvic CT, chest CT, pelvic MRI, EUS (optional), and sigmoidoscopy. The response was determined based on the findings of rectal examination, sigmoidoscopy, and MRI.

**Figure 2:**
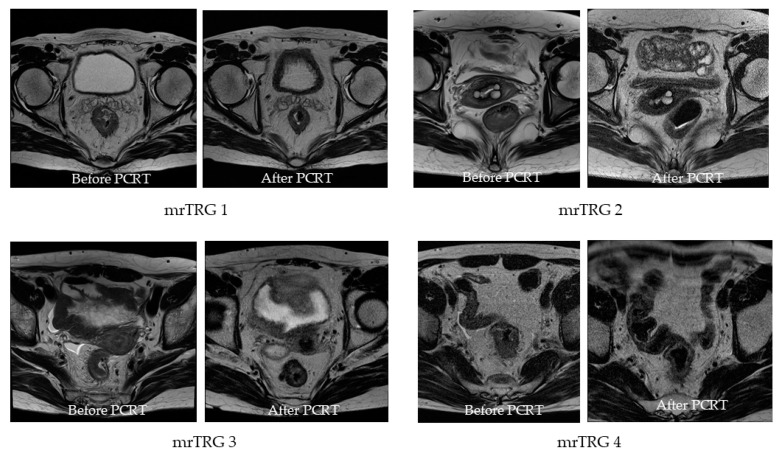
Tumor response after preoperative chemoradiotherapy (PCRT) with MRI as mrTRG. mrTRG, tumor regression grade evaluated with MRI; mrTRG1, complete regression; mrTRG2, near complete regression; mrTRG3, moderate regression; mrTRG4, minimal to no regression.

Clinical tumor response was evaluated with MRI as mrTRG score of 1 to 4 according to proportion of remained tumor signal and fibrosis: 1, complete regression (absence of tumor signal and barely visible treatment related scar); 2, near complete regression (predominant low signal intensity fibrosis with no obvious residual tumor signal); 3, moderate regression (low signal intensity fibrosis predominates but there are obvious areas of intermediate signal intensity); 4, minimal to no regression (small areas of low signal intensity fibrosis or mucin but mostly tumor or intermediate signal intensity, same appearances as original tumor) (Figure 2).

Resection of the tumor was performed 6 to 8 weeks after the completion of PCRT. Among patients with good response to PCRT showing CR or near-CR for both T and N stages, the surgical method of LE or RR was determined by the surgeon and patients, depending on factors, such as age, medical condition, and socioeconomic status. Patients were informed of the pros and cons of each procedure. Patients who were reluctant to receive a temporary or permanent stoma were more likely to choose LE, and surgeons offered LE to patients at elevated risk due to longer general anesthesia and postoperative complications due to medical comorbidities. Radical surgical resection was performed according to TME. For LE, transanal local excision and transanal minimally invasive surgery and/or full-thickness excision was performed.

Tumor response was assessed by a pathologist specializing in colorectal malignancy. A tumor regression grading system was used to determine the response of the primary tumor according to the proportion of tumor cells and fibrosis in resected specimens, as suggested by the Gastrointestinal Pathology Study Group of the Korean Society of Pathologists [19]. Pathologic staging after RR was determined according to the seventh American Joint Committee on Cancer Staging System. Patients with an indeterminate tumor regression grade or inability to confirm recurrence status were excluded from the study. Immediate salvage RR was strongly recommended to patients diagnosed with ≥ypT2 stage after LE. In addition, patients with deep submucosa invasion, lymphovascular invasion, perineural invasion, tumor budding, and margin involvement were also recommended to undergo salvage operations.

All medically fit patients underwent adjuvant chemotherapy after RR with PCRT. Adjuvant chemotherapy consisted of either four cycles of FL monthly or six cycles of capecitabine. FOLFOX (8 cycles, 5-fluorouracil and oxaliplatin) was delivered to patients with ypT3-4 or N+ disease.

### 2.3. Postoperative Surveillance and Recurrence

**Figure 3:**
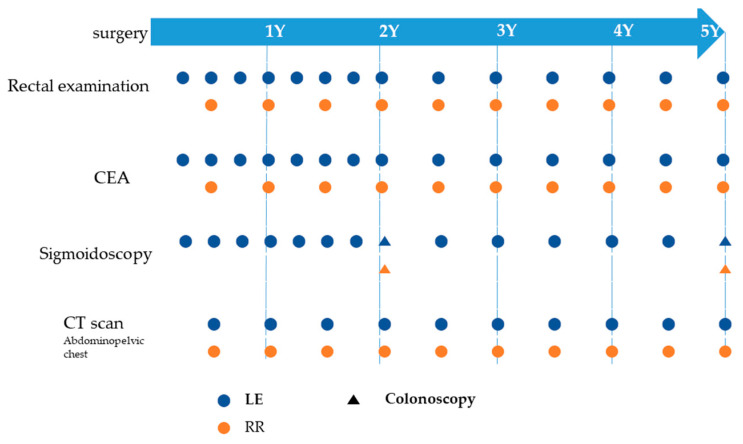
Scheme of surveillance protocol. Colonoscopy was performed every 2 or 3 years. RR, radical resection; LE, local excision; CT, computed tomography.

Patients in the LE group got surveillance work-up as follows: Physical examination with a digital rectal examination, checkup of laboratory test results, and sigmoidoscopy were done every 3 months for the first 1 to 2 years and every 6 months for the next 3 to 4 years. Full colonoscopy was performed every 2 to 3 years. CT scan of the abdominopelvic and chest regions was performed every 6 months for 5 years. Those in the RR group underwent physical examination, laboratory tests, abdominopelvic and chest CT scans every 6 months for 5 years, while a full colonoscopy was performed every 2 to 3 years (Figure 3).

Clinical, radiologic, or endoscopic evidence of intraluminal tumor in contiguous areas to the primary resection site, tumor within the mesorectum or rectal wall after excision was defined as local recurrence. In contrast, distant metastasis was defined as the dissemination of the tumor to the peritoneal surface or tumor presence in a distant organ.

### 2.4. Statistical Analysis

Analyses of clinicopathologic characteristics of categorical variables and continuous variables were conducted using the chi-squared test and t-test, respectively. The Kaplan-Meier method with log-rank test was used to determine the RFS and OS. RFS was measured from the date of resection to the date of the identification of the first recurrence. Overall survival was identified as duration from date of resection to any cause of death. A multivariable analysis with the Cox proportional hazards model was used to compare risk factors associated with RFS and OS. *p*-values of less than 0.05 were considered statistically significant. All analyses were conducted using IBM SPSS Statistics ver. 21.0 (IBM Co., Armonk, NY, USA).

## 3. Results

### 3.1. Clinicopathological Characteristics

The clinicopathological features of the patients are shown in Table 1. Among the 520 patients, 78 patients underwent LE and 442 underwent RR. Patients in the LE group was older than RR group. In the LE group, patients with initial cT1-2 or cN(−) were significantly more than in the RR group. Among LE group, 61 (78.2%) patients received transanal excision and 17 (21.8%) were treated with transanal minimally invasive local excision. In the RR group, sphincter saving resection was done for 348 (78.7%) patients. As preoperative concurrent chemotherapy, 19 patients received oxaliplatin-combination regimens (4 with capecitabine, 3 with fluorouracil, and 12 with tegafur/gimeracil/oteracil (TS-1)). Temozolomide was administered to three patients based on the clinical study setting.

More patients in the RR group (85.5%) underwent adjuvant chemotherapy (CTx) after surgery than the LE group (44.9%). Among patients (33, 7.5%) in the RR group with positive lymph nodes (LNs), a mean of 26 LNs were excised, and an average of 1.92 malignant LNs were detected among the resected LNs. LN metastasis occurred in 22 of 307 patients with ypT0 (7.2%) and 11 of 135 patients with ypTis-T1 (8.1%). Lymphovascular invasion (LVI) was identified in four patients (0.9%) and perineural invasion (PNI) in 28 patients (6.3%). Circumferential margin (CRM) was positive (<1 mm) in one patient (0.2%). Deep resection margin was involved in two patients (2.6%) in the LE group.

In the LE group, surgical complications occurred in two patients (2.5%). Perirectal inflammation requiring conservative treatment developed in one patient, and one patient had a pelvic abscess and subsequent stoma formation. In the RR group, anastomotic leakage and pelvic abscesses occurred in 15 patients (3.4%).

### 3.2. Oncologic Outcomes: Recurrence and Survival

In the LE group, seven (8.9%) patients experienced recurrence, three (42.9%) showed local recurrence, and four (57.1%) showed recurrence in the distant LN (1 patient, 14.3%) and lung (3 patients, 42.9%). In the LE group, nine patients were recommended to undergo radical resection immediately after local excision due to unfavorable risk factors such as submucosal invasion >2000 μm, lymphovascular invasion, perineural invasion, or indeterminate or positive resection margin; however, only one did so. Among these nine patients, three experienced recurrences, including two luminal recurrences in patients who did not undergo radical resection. Distant lymph node (inguinal LN) metastasis occurred in the patient who underwent immediate radical resection.

In the RR group, 43 (9.7%) patients experienced recurrence, three (7.0%) with local recurrence, and 40 (93.0%) with distant metastasis. Single-organ distant metastasis was detected in 37 patients in the lung (20 patients, 46.5%), liver (8 patients, 18.6%), bone (4 patients, 9.3%), and distant LN (4 patients, 9.3%). Three patients had multiple organ metastasis found in the brain and lung (1 patient, 2.3%), distant LN and liver (1 patient, 2.3%), and distant LN and lung (1 patient, 2.3%) (Table 2). Recurrence was noted in the LN, lung, liver, bone, brain, and ovary in the RR group. The time to recurrence among patients who experienced recurrence was 27.64 ± 19.70 months in the LE group and 25.69 ± 27.47 months in the RR group (*p* = 0.858). Patients in the LE group with recurrence all underwent adjuvant CTx. Two patients underwent salvage surgery, one patient underwent additional transanal excision, and the other underwent radical resection. Among the patients who received salvage surgery, the patient who underwent radical resection experienced distant lymph node metastasis 8 months after salvage surgery and showed disease progression. The patient treated with re-do transanal excision remained tumor-free through the final surveillance.

**Figure 4:**
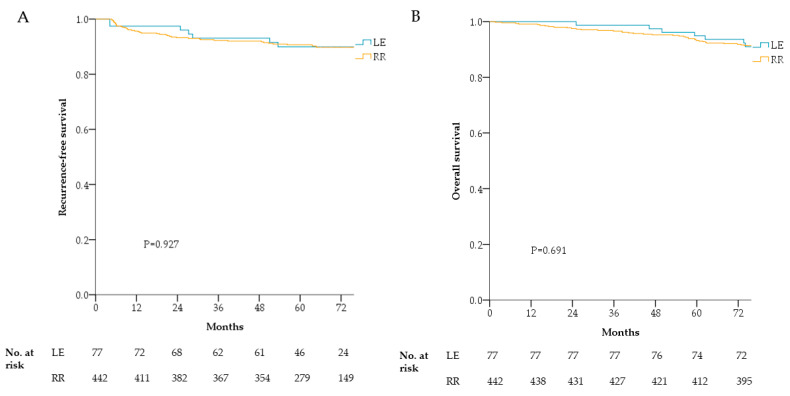
Oncologic outcomes according to surgical treatment of ypT0-1 rectal cancer after preoperative chemoradiotherapy. Recurrence-free survival (**A**) and overall survival (**B**). RR, radical resection; LE, local excision.

The 5-year RFS rate was higher in the RR group (94.7%) than in the LE group (98.0%), but it was not statistically significant (*p* = 0.927). The 5-year OS rates were similar in the LE (94.9%) and RR (93.7%; *p* = 0.691) groups (Figure 4).

There were no factors associated with RFS in ypT0-1 cancer, except for the cT stage before PCRT (Table 3).

When the subgroup analysis was performed in the RR group, LN metastasis was the only factor associated with RFS (Table 4).

None of the factors showed a statistically significant association with OS.

However, in the subgroup analysis of RR, LN metastasis and age were significantly associated with OS (Table 5).

## 4. Discussion

In this study, we identified that the RFS and OS did not differ according to the extent of resection, categorized into local and radical excision, in patients with ypT0-1 after preoperative chemoradiotherapy. Although LN metastasis was only a significant factor associated with RFS and OS in the RR group, we could not find any risk factors for RFS and OS in the overall cohort.

The down-staging effect of PCRT led the patients and surgeons to consider a less invasive way to sufficiently treat rectal cancer with better function postoperatively, without compromising oncologic outcomes. Organ-preserving strategies, such as LE of the tumor or close monitoring of the disease progression without any interventions (WW approach) could be applied for rectal cancer patients with clinical good response to PCRT. In terms of postoperative quality of life, the patients can live without transient/permanent stoma and avoid a higher rate of surgical complications [6,7,9].

In previous studies, long-term oncologic outcomes between LE and RR were shown to have no statistically significant difference in terms of local recurrence, RFS, and OS rate in patients with good response to PCRT [11,20]. Our study also showed consistent results when comparing the 5-year RFS and OS of LE and RR, showing 98.0% vs. 94.7% and 94.9% vs. 93.7%, respectively, with no statistical significance. Therefore, by undergoing LE instead of RR, patients can potentially avoid unnecessary radical surgery and the associated morbidity and mortality with comparable long-term treatment results to RR.

For the successful application of LE in rectal cancer with good response to PCRT, proper patient selection is critical [21].

Careful reevaluation is performed with state-of-the-art MRI, but it is inevitably less accurate than pathologic staging [22]. Even with pelvic MRI, which is considered superior to EUS, limitations remain in predicting T stage and the relationship of the tumor with mesorectal fascia [23]. It becomes more challenging to differentiate fibrosis from residual viable tumors especially after PCRT. Studies report a poor correlation of post-PCRT imaging and pathologic results, showing an accuracy of 47% to 52% in T stage and 64% to 68% in N stage, with an overall accuracy reported to be approximately 68% to 72% in diffusion-weighted imaging [15,24,25,26]. Detecting the reduction of the tumor metabolism after PCRT is continuously studied to predict the pathologic response of the treatment by establishing a response predictive model with PET-CT [27]. However, the accuracy in confirming the clinical response is reported to be only 44% [25]. Based on the limitations of this study, it is difficult to determine surgical strategies depending on post-treatment clinical stage. Local excision, therefore, can be considered as one option for response assessment after PCRT. The LN metastasis status still could not be evaluated via local excision, but we can perform careful surveillance for them if oncologic outcomes after local excision are not compromised.

Taken together, the results of this study showed that LN metastasis is the strongest associated factor with RFS, even in patients who showed good response to PCRT. Approximately 5% of the patients with ypT0 are reported to have positive LNs at the pathological examination [1,2,9,28]. In our study, 7.2% (22 of 307 RR patients) of ypT0 patients were noted to have LN metastases in the final pathology. The lack of an accurate modality to evaluate LN status in patients undergoing local excision leaves an incomplete understanding of the oncologic outcome of LE, and studies are still ongoing in this regard.

Performing adjuvant therapy in patients with CR using PCRT also lacks consensus. In our study, patients underwent chemotherapy based on treatment guidelines with some modifications according to the patient’s general condition and physician’s preference. The decision to undergo adjuvant chemotherapy was determined by the initial clinical tumor stage regardless of the final pathologic state. However, much fewer patients treated with LE received adjuvant chemotherapy compared with those in the RR group. The LE group included more patients who were reluctant to undergo aggressive treatment, and this might have influenced the adjuvant chemotherapy receipt rate. In addition, the LE group was older and more fragile relative to treatment, although these were not contraindications for treatment. Regardless of these disadvantageous clinical features, RFS and OS were not inferior in the LE group. Although the number of patients who received adjuvant chemotherapy was significantly less in the LE group, there was no difference in the RFS between the two groups. Long-term oncologic outcomes and standard treatment guidelines on adjuvant chemotherapy in ypT0 are scarce. However, recent studies report no significant benefit of adjuvant chemotherapy in rectal cancer with good response to PCRT. Several studies involving patients with ypT0-2N0 rectal cancer treated with adjuvant chemotherapy showed no influence on RFS [27,29,30]. In our study, adjuvant chemotherapy in ypT0 patients had no statistical significance in the RFS and OS in both groups. However, further prospective randomized studies with a larger sample size are needed to validate whether there are no additional oncologic benefits.

Additionally, the organ-preservation strategy is evolving and becoming more common than LE. The WW strategy is actively being introduced. Despite having a lower morbidity rate, considerable rates of anorectal pain, wound dehiscence, and readmission to hospitals are reported in patients who underwent LE, and is worse after undergoing PCRT [11,31,32]. Clinical assessment strategies for tumors are constantly developing, and accuracy in the selection of patients who can be suitable candidates for less invasive treatment is improving, but it is not satisfactory yet. Although avoiding LE in patients who show CR to PCRT may be a safer option to avoid any surgical complications [33,34], the benefit of LE, which is the pathologic confirmation of ypT status compared to WW strategy, needs to be reappraised.

It has been more than 15 years since the organ-preserving strategy was introduced, but there have been no standard treatment guidelines for LE. Clinicians can often be hesitant because of the remaining risk of insufficient evaluation of the cancer, which leads to irreversible detrimental results despite widespread increased interest in organ-preserving strategies [35,36,37]. However, it is undeniable that more conservative ways are gaining popularity to preserve organs with less complications. Delaying the establishment of a standardized protocol for LE or WW strategy will worsen the variation of patient selection and evaluation of long-term treatment goals, such as follow-up methods or period and further treatment strategies, including adjuvant chemotherapy and salvage surgery after local recurrence.

There are some limitations to this study. First, it was a retrospective study using a single-center database with heterogeneous patient features. It was inevitable that the RR group had more patients with advanced disease who underwent adjuvant chemotherapy. Additionally, as patients with poor general condition or with more comorbidities are considered to undergo less invasive treatment, the average age of the patients was higher in the LE group. However, none of the factors showed statistical significance in analyzing the RFS and OS. Indeed, the pre-PCRT clinical stage, which is an important risk factor for RFS, is difficult to evaluate correctly and might influence risk factor analysis, although we re-evaluated the MRI-based clinical stage for this study. Second, due to the extended period of the study, inter-observer variability in the interpretation of imaging and differences in chemotherapy regimens could have been present, but diagnostic modalities in our center have always been up to date, and treatment was in line with standardized guidelines.

## 5. Conclusions

Despite these limitations, this study included many patients who underwent PCRT with standardized surgery at a qualified institution with more than 10 years of follow-up time and will sufficiently serve as reference data for further studies. LE and RR are comparable in terms of RFS and OS for patients who had ypT0-1 rectal cancer after preoperative chemoradiotherapy. A prospective study with a larger sample size and refined treatment protocols will better elucidate the best way forward for organ-preserving strategies. However, it is difficult to conduct large-scale trials for this issue; thus, a well-matched controlled study may be an alternative.

## Figures and Tables

**Table 1:** Clinicopathological characteristics.

Characteristics	Local Excision (*n* = 78)	Radical Resection (*n* = 442)	*p*-Value
Sex (%)			0.557
Male	44 (56.4)	265 (60.0)	
Female	34 (34.6)	177 (40.0)	
Age, years, mean ± SD	62.9 ± 11.3	58.5 ± 10.2	0.001
Concurrent chemotherapeutic regimen (%)			0.02
Capecitabine	26 (33.3)	196 (44.3)	
FL	49 (62.8)	198 (44.8)	
Others	1 (1.3)	22 (5.0)	
unknown	2 (2.6)	26 (5.9)	
cT stage (%)			<0.001
1-2	55 (70.5)	76 (17.2)	
3-4	23 (29.5)	366 (82.8)	
cN stage (%)			<0.001
cN (−)	41 (52.6)	58 (13.1)	
cN (+)	37 (47.4)	384 (86.9)	
Sphincter saving resection *		348 (78.7)	
ypT stage			0.89
ypT0	54 (69.2)	307 (55.2)	
ypTis	16 (20.5)	103 (23.3)	
ypT1	8 (10.3)	32 (7.2)	
ypN stage *			
ypN(−)	N/A	409 (96.9)	
ypN(+)	N/A	33 (7.5)	
Adjuvant chemotherapy			<0.001
No	43 (55.1)	64 (14.5)	
Yes	35 (44.9)	378 (85.5)	
Adjuvant chemotherapy regimen			<0.001
Capecitabine	9 (25.7)	150 (39.6)	
FL	23 (65.7)	183 (48.4)	
Oxalplatin-combination	0	18 (4.1)	
Unknown	5 (8.6)	38 (8.6)	
Follow-up duration (months)	66.9 ± 28.7	71.7 ± 33.2	0.244

SD standard deviation, IV intravenous, FL 5-fluorouracil and leucovorin. Values are presented as number (%) or mean ± SD unless otherwise indicated. * Only evaluated in the radical resection group.

**Table 2:** Recurrence site in ypT0-1 rectal cancer treated with preoperative chemoradiotherapy according to the surgical method.

Recurrence Site	Group		*p*-Value
	Local Excision (%)	Radical Resection (%)	
Local recurrence	3 (42.9)	3 (7.0)	0.029
Distant recurrence	4 (57.1)	40 (93.0)	
Total	7	43	
Single organ	7 (100.0)	40 (93.0)	0.630
Multiple organ	0 (0.0)	3 (7.0)	
Total	7	40	

**Table 3:** Factors associated with recurrence-free survival in ypT0-1 rectal cancer treated with preoperative chemoradiotherapy.

Variable	Hazard Ratio	95% Confidence Interval	*p*-Value
Resection group			
Local excision	1		
Radical resection	0.687	0.265–1.780	0.440
Clinical T stage			
cT1-2	1		
cT3-4	3.011	1.170–7.745	0.022
Clinical N stage			
cN(−)	1		
cN(+)	0.514	0.250–1.056	0.070
Sex			
Male	1		
Female	1.196	0.693–2.094	0.531
Adjuvant chemotherapy			
No	1		
Yes	1.355	0.600-3.061	0.465

**Table 4:** Factors associated with recurrence-free survival in ypT0-1 rectal cancer treated radical resection after preoperative chemoradiotherapy.

Variable	Hazard Ratio	*p*-Value	Hazard Ratio	95% Confidence Interval	*p*-Value
Age	0.985	0.313			
Clinical T stage		0.095			0.145
cT1-2	1		1		
cT3-4	2.719		2.402	0.739–7.806	
Clinical N stage		0.323			
cN(−)	1				
cN(+)	0.678				
Sex		0.212			
Male	1				
Female	1.463				
Sphincter preservation		0.114			0.142
No	1		1		
Yes	0.590		0.611	0.317–1.178	
ypN stage		<0.001			<0.001
ypN (−)	1		1		
ypN (+)	4.855		4.884	2.451–9.732	
Lymphovascular invasion	0.771	0.315			
Perineural invasion	0.760	0.705			
Adjuvant chemotherapy		0.368			
No	1				
Yes	1.605				

**Table 5:** Factors associated with overall survival in ypT0-1 rectal cancer treated with radical resection after preoperative chemoradiotherapy.

Variable	Hazard Ratio	*p*-Value	Hazard Ratio	95% Confidence Interval	*p*-Value
Age	1.047	0.003	1.046	1.104–1.080	0.004
Clinical T stage		0.376			
cT1-2	1				
cT3-4	1.471				
Clinical N stage		0.969			
cN(−)	1				
cN(+)	0.984				
Sex		0.553			
Male	1				
Female	0.839				
Sphincter preservation		0.682			
No	1				
Yes	0.872				
ypN stage		<0.001			<0.001
ypN (−)	1		1		
ypN (+)	3.850		4.302	2.187–8.462	
Lymphovascular invasion	2.392	0.389			
Perineural invasion	1.109	0.975			
Adjuvant chemotherapy		0.161			0.347
No	1		1		
Yes	1.609		0.704	0.339–1.463	

## Data Availability

The data presented in this study are available on request from the corresponding author. The data are not publicly available due to the institutional policy.
